# Early Deficits in Glycolysis Are Specific to Striatal Neurons from a Rat Model of Huntington Disease

**DOI:** 10.1371/journal.pone.0081528

**Published:** 2013-11-26

**Authors:** Caroline Gouarné, Gwenaëlle Tardif, Jennifer Tracz, Virginie Latyszenok, Magali Michaud, Laura Emily Clemens, Libo Yu-Taeger, Huu Phuc Nguyen, Thierry Bordet, Rebecca M. Pruss

**Affiliations:** 1 Trophos, Parc Scientifique de Luminy, Luminy Biotech Entreprises, Marseille, France; 2 Institute of Medical Genetics and Applied Genomics, University of Tuebingen, Tuebingen, Germany; 3 Center for Rare Diseases, University of Tuebingen, Tuebingen, Germany; Oregon Health & Science University, United States of America

## Abstract

In Huntington disease (HD), there is increasing evidence for a link between mutant huntingtin expression, mitochondrial dysfunction, energetic deficits and neurodegeneration but the precise nature, causes and order of these events remain to be determined. In this work, our objective was to evaluate mitochondrial respiratory function in intact, non-permeabilized, neurons derived from a transgenic rat model for HD compared to their wild type littermates by measuring oxygen consumption rates and extracellular acidification rates. Although HD striatal neurons had similar respiratory capacity as those from their wild-type littermates when they were incubated in rich medium containing a supra-physiological glucose concentration (25 mM), pyruvate and amino acids, respiratory defects emerged when cells were incubated in media containing only a physiological cerebral level of glucose (2.5 mM). According to the concept that glucose is not the sole substrate used by the brain for neuronal energy production, we provide evidence that primary neurons can use lactate as well as pyruvate to fuel the mitochondrial respiratory chain. In contrast to glucose, we found no major deficits in HD striatal neurons’ capacity to use pyruvate as a respiratory substrate compared to wild type littermates. Additionally, we used extracellular acidification rates to confirm a reduction in anaerobic glycolysis in the same cells. Interestingly, the metabolic disturbances observed in striatal neurons were not seen in primary cortical neurons, a brain region affected in later stages of HD. In conclusion, our results argue for a dysfunction in glycolysis, which might precede any defects in the respiratory chain itself, and these are early events in the onset of disease.

## Introduction

Huntington disease (HD) is a hereditary neurodegenerative disorder caused by a CAG repeat extension in the coding region of the huntingtin gene, leading to striatal atrophy which later expands to the cerebral cortex and other subcortical brain regions [1,2]. Clinically, the disease is characterized by psychiatric symptoms, movement disorders, progressive dementia and also by pronounced weight loss despite sustained caloric intake [3] supporting to the hypothesis of impaired ATP synthesis in HD [4,5]. This was further confirmed by the detection of significant alterations in the glucose concentration by brain imaging [6-8] and in the concentration of energetic metabolites (mainly N-acetylaspartate, glutamine/glutamate, and lactate) in brain or in the cerebrospinal fluid of HD patients [9-15]. Whether this results from reduced mitochondrial ATP synthesis and/or reduced glycolytic ATP levels is not known. The observation of a severe reduction in the activity of the mitochondrial respiratory chain complexes II/III and a milder reduction in the activity of complex IV in the caudate/putamen from post-mortem brain samples suggested that mitochondrial abnormalities may underlie HD pathogenesis [16-18]. However, whether respiratory chain impairment is the cause or the consequence of neuronal loss in HD remains unclear, since such defects were not observed in pre-symptomatic patients [19,20]. 

To further address the precise nature and the role of metabolic and mitochondrial dysfunction in HD, studies were performed in genetic models of HD, particularly in mice expressing full-length mutant huntingtin (fl-mHtt). As observed in pre-symptomatic and early HD patients, no major impairment in the enzymatic activity of the mitochondrial respiratory chain complexes I–IV was evidenced in either the striatum or the sensorimotor cortex of these mice [19]. By contrast, deficits in respiration rate and ATP production reported in ST*Hdh* Q111 striatal cell lines derived from knock-in mice with 111 CAG repeats introduced into the mouse HTT homologue *Hdh* [21-23]. However, the impairment could not be assigned to defects in individual respiratory complexes in these cells. Moreover, differences in mitochondrial respiratory rates were no longer present when using isolated mitochondria from the same cell lines [24], suggesting that detection of some mitochondria deficits may only be detected in intact cells. In that sense, Oliveira and colleagues measured mitochondrial respiratory rates in intact, non-permeabilized, primary striatal neurons from *Hdh*150 knock-in mice and did not find any deficit under resting condition when compared to wild type controls [25]. However, when challenged with an energy-demanding stimulus (NMDA-receptor activation) and incubated in pyruvate-based media to accentuate mitochondrial metabolism, *Hdh*150 neurons were more vulnerable to calcium overload than neurons from their wild-type littermates [26]. These results highlight the importance of assessing mitochondrial function in the cellular environment including both neuronal cell bodies and neurites. It is also important to survey the use of various glycolytic and oxidative substrates to better understand how cells cope with metabolic demands *in situ*. 

To pursue these questions, we investigated mitochondrial respiration in specific primary neuronal subpopulations cultured from a new HD transgenic rat model expressing fl-mHtt and all regulatory elements integrated from a bacterial artificial chromosome (BACHD rats). These rats display a robust, earlier onset and faster progressive HD-like phenotype even in heterozygous transgenic rats compared to previously described HD rats expressing a mHtt fragment [27]. Oxygen consumption rates and anaerobic glycolysis were measured using a variety of substrates *in situ*, directly in the culture well, preserving the neuronal network integrity.

## Materials and Methods

### Ethics statement

All experiments were approved and performed in accordance with internal institutional guidelines; Trophos is an accredited institution for animal experimentation in France (French ministry of Agriculture, Agreements No. B-13-055-15) and in strict accordance with national and European regulations (Directive 86/609/EEC of the European Economic Community regarding the protection of animals used for experimental and other scientific purposes). All efforts were made to avoid or minimize animal suffering and to reduce and refine the experimentation.

### Animals

Male heterozygous transgenic BACHD rats (HD) expressing high levels of fl-mHtt protein containing 97 CAG/CAA repeats (line LY.005) [27] were bred with female wild type (WT) rats obtained from Elevage Janvier (Le Genest Saint Isle, France). The rats were housed in a controlled environment (room temperature 22°C ± 2; reverse 12h light dark cycle, 50% ± 5 humidity) with food and water available ad libitum. Pregnant female rats were euthanized at embryonic day 17 (E17) by gradual fill CO_2_ overdose. Rats were placed in a hermetic box then exposed to a mixture of O_2_/CO_2_ (40-60% respectively) until sleep was induced. The % CO_2_ was then progressively increased up to 100% while O_2_ was decreased (0.5L/min every 30 sec. for 3 min up to 5L/min) and then maintained for 4-5 min. 

### Genotype identification

Rat embryos from a single pregnant female rat were used for each independent experiment. All embryos were genotyped to identify WT and HD animals. During genotyping, brains were maintained in Hibernate® conservation medium (BrainBits) at 4°C. The embryos were genotyped with a PCR-based assay using DNA from tail tissue. The mHTT gene product was detected using the primers 5’-ATGGCGACCCTGGAAAAGCTG-3’ and 5‘-AGGTCGGTGCAGAGGCTCCTC-3’ (Eurofins). PCR conditions were: 30 cycles at 94°C (30 s), 60°C (30 s), 72°C (1 min).

### Primary neuronal cultures

E17 primary striatal neurons were prepared by an adapted method previously described [28]. Briefly, striata were thoroughly minced into 1 mm sections, washed in HBSS medium (Invitrogen) complemented with 7 mM HEPES (Invitrogen) and 0.45% glucose (w/v) (Sigma) and incubated for 15 min at 37°C with 0.25% of trypsin (Invitrogen). The tissues were mechanically dissociated by several pipetting and centrifugation steps (5 min at 250 x g). Primary striatal neurons were seeded onto poly-ornithine (Invitrogen) and laminin (BD) coated Seahorse 24-well plates at a density of 150,000 cells/well in Neurobasal™ media (Invitrogen), complemented with 2% B-27 supplement (Gibco) and 1 mM pyruvate (Invitrogen). The two cell populations (WT and HD) were seeded in different wells of the same plate with 4 to 8 replicates per plate depending on the protocol. The cells were incubated for 7 days at 37°C in a humidified incubator in an atmosphere of 95% O_2_, 5% CO_2_. E17 primary cortical neurons were prepared using a similar protocol and seeded at a density of 110,000 cells/well. Striatal and cortical neuronal culture purity was controlled by immunofluorescence using NeuN antibody (Millipore) to identify neurons, GFAP (Millipore) for astrocytes and F4/80 (Santa Cruz) for microglial cells. Both striatal and cortical neuronal cultures were 98% pure. The presence of mHtt, already in the form of aggregates, was confirmed in primary striatal and cortical neuronal cultures derived from HD rats (see in Protocol S1 and Figure S1).

### Oxygen consumption rate and extracellular acidification rate measurements-

Cellular oxygen consumption and extracellular acidification rate (ECAR) reflecting lactate release were concomitantly measured using a XF24 extracellular flux analyzer (Seahorse Bioscience) as previously described [29]. 

### Respiration experiments in substrate-rich medium

**Figure 1:**
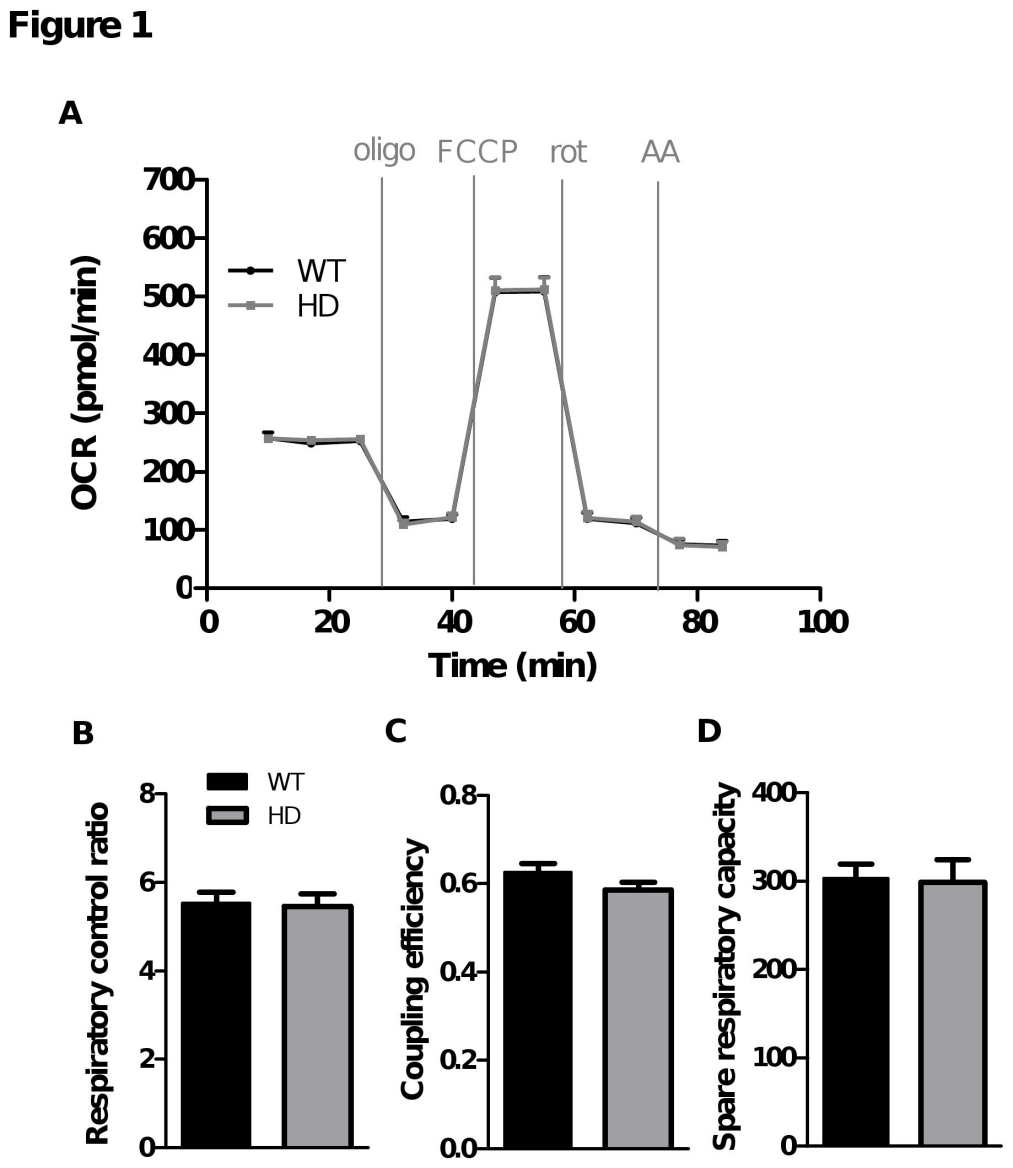
No differences in respiratory parameters are detected between HD and WT striatal neurons incubated in substrate-rich medium. OCR measurements were performed in bicarbonate- and HEPES-free DMEM containing 25 mM glucose complemented with pyruvate (1 mM) and began 10 min after cell plate installation in the Seahorse analyser. A) OCR was measured sequentially in basal conditions (time points 1-3), after injections of oligomycin (oligo; time points 4 and 5), FCCP (time points 6 and 7), rotenone (time points 8 and 9) and then antimycin A (AA; time points 10 and 11) at the indicated times. Data shown are mean ± SEM from a representative experiment (eight replicates). Respiratory parameters were derived from OCR measured during each phase of the experiment as shown in A: B) respiratory control ratio = [FCCP rate (time point 6) / oligo rate (time point 5)]; C) coupling efficiency = [basal rate (time point 3) – oligo rate (time point 5)] / basal rate (time point 3); D) spare respiratory capacity = [FCCP rate (time point 6) – basal rate (time point 3)]. Respiratory parameters shown in panels B-D are mean ± SEM calculated from all replicates (eight replicates in each of three independent experiments).

Experiments in substrate-rich media were performed using Dulbecco’s Modified Eagle’s Medium (DMEM) containing 25 mM glucose without bicarbonate (Invitrogen) and complemented with 1 mM fresh pyruvate (assay medium). One hour before the start of the respiration measurements, the original culture medium was replaced by assay medium and plates were placed into the XF analyzer calibrated at 37°C. After 10 min of equilibration, OCR was measured for 1.5 min to establish a baseline rate. The medium was then gently mixed for 2 min followed by a 3 min pause to restore normal oxygen tension in the microenvironment surrounding the cells. After baseline measurements, oligomycin (final concentration 0.5 µg/ml) (Sigma) was injected into each well in order to inhibit mitochondrial ATP synthase to determine proton leak-dependent OCR (State 4o). After this, the uncoupling agent carbonilcyanide *p*-triflouromethoxyphenylhydrazone (FCCP; final concentration 2 µM) (Sigma) was injected to determine maximal OCR (state 3u). Finally, rotenone (final concentration 75 nM) (Sigma) and antimycin A (final concentration 150 ng/ml) (Sigma) were injected to inhibit complex 1 and complex 3 respectively and to completely abolish mitochondrial respiration. After each injection step, two measurement cycles (measurement + mix + pause) were performed (Figure 1) and after rotenone and antimycin A injection, 4 measurement cycles were performed. Preliminary experiments determined the optimal concentration of these agents to optimally inhibit or stimulate respiration without inducing toxicity. 

### Substrate-dependent respiration experiments

To assay the ability of different substrates to support respiration, experiments were designed using a simple substrate-free modified Krebs Henseleit Buffer (KHB) (NaCl 111 mM, KCl 4.7 mM, MgSO_4_ 2 mM, Na_2_HPO_4_ 1.2 mM, pH 7.4), complemented with 2 mM GlutaMAX™ (Invitrogen). One hour before the start of the assay, the original culture medium was replaced by KHB and the plates were placed in an incubator at 37°C without CO_2_ and then in the XF analyzer. After 10 min of equilibration in the instrument, OCR was measured for 1.5 min to establish a substrate free basal rate. Medium was then gently mixed for 2 min followed by a 3 min pause to restore normal oxygen tension in the microenvironment surrounding the cells. After baseline measurements, the substrates D-glucose (Sigma), pyruvate (Sigma) or lactate (Sigma) were injected and OCR was measured for 1.5 min followed by injections of oligomycin, FCCP and rotenone plus antimycin A as described above. After each injection, two or three measurement cycles (measurement + mix + pause) were performed. 

### Respiration parameters calculation

When oxygen consumption rate measurements ended, cells were incubated for 20 minutes with 2 µg/ml calcein-AM to measure the relative neuronal cell density and viability in each well. Only experiments with a similar cell density based on calcein fluorescence between WT and HD (Student’s t test p > 0.05) were validated and analyzed. Respiration parameters were calculated from data obtained during the steps of OCR measurement. For basal respiration and oligomycin respiration, the last OCR measurement of the cycle was selected for calculation. For FCCP the first OCR measurement of the cycle was selected to avoid the effect of substrates depletion on maximal OCR. For further details please refer to the legend of the respective figures. Because non-mitochondrial oxygen consumption rate (measured after antimycin A injection) was overestimated due to the fact that FCCP injection activated non-mitochondrial oxidase and because the rate was negligible (according to preliminary assays), we decided to not remove non-mitochondrial OCR from all measurements. Cell respiratory control ratio (RCR) was calculated by dividing the maximal rate (uncoupled) by the oligomycin rate (proton leak). Spare respiratory capacity was calculated by subtracting the basal rate from the maximal (uncoupled) rate. Coupling efficiency was calculated by dividing the fraction of basal mitochondrial oxygen consumption used for ATP synthesis (basal rate minus oligomycin rate) by the basal rate. The substrate response was determined by dividing the OCR obtained after substrate addition (glucose, pyruvate, lactate, or combinations) by the substrate-free basal rate in KHB. 

### Statistical analysis

Data are expressed as means ± SEM. Comparisons between two groups were performed using an unpaired Student’s t test. Comparisons of several groups or differences between treatments and experimental groups were conducted using a one way or two way analysis of variance (ANOVA) followed by Dunnett’s post test. A p-value of less than 0.05 was considered statistically significant. 

## Results

### Characterization of WT and HD striatal neuron respiration in substrate-rich medium

E17 primary striatal neurons from HD and WT rat embryos were cultured for 7 days in Neurobasal™ medium and then placed in DMEM with similar concentrations of amino acids, vitamins and glucose (25 mM) to measure OCR. Their response to the respiration analysis paradigm in substrate rich medium is shown in Figure 1A. Basal OCR is strongly controlled by ATP turnover and to a lesser extent by substrate oxidation and proton leak [30]. Addition of oligomycin induced a decrease in OCR and under this condition (state 4o), respiration is strongly controlled by proton leak kinetics and partially by substrate oxidation [30]. In contrast, OCR sensitive to oligomycin is strongly driven by the proton flux accompanying ATP synthesis. Addition of the uncoupler (FCCP) caused an increase in OCR until the maximum respiration rate was reached (state3u). This respiratory rate reflects the maximal respiratory chain activity as well as the maximal substrate oxidation rate that is achievable [30]. Finally, addition of rotenone (complex 1 inhibitor) and antimycin A (complex 3 inhibitor) totally blocked the respiratory chain and the residual OCR represented non-mitochondrial respiration, probably driven by NADPH oxidase activity [30]. In these experiments performed in substrate rich DMEM, no differences in OCR or respiratory parameters were detectable between WT and HD striatal neurons (Figure 1). Similarly, HD striatal neurons showed no deficits in RCR, which detects substrate oxidation efficiency and respiration due to proton leak, but not driven by ATP synthesis (Figure 1B), coupling efficiency, which is strongly controlled by ATP turnover and proton leak (Figure 1C) or spare respiratory capacity, which reflects the capacity of the respiratory chain to respond to an increase in energy demand and the ability of substrates to provide fuel (Figure 1D). 

### Glucose supported respiration in striatal neurons

**Figure 2:**
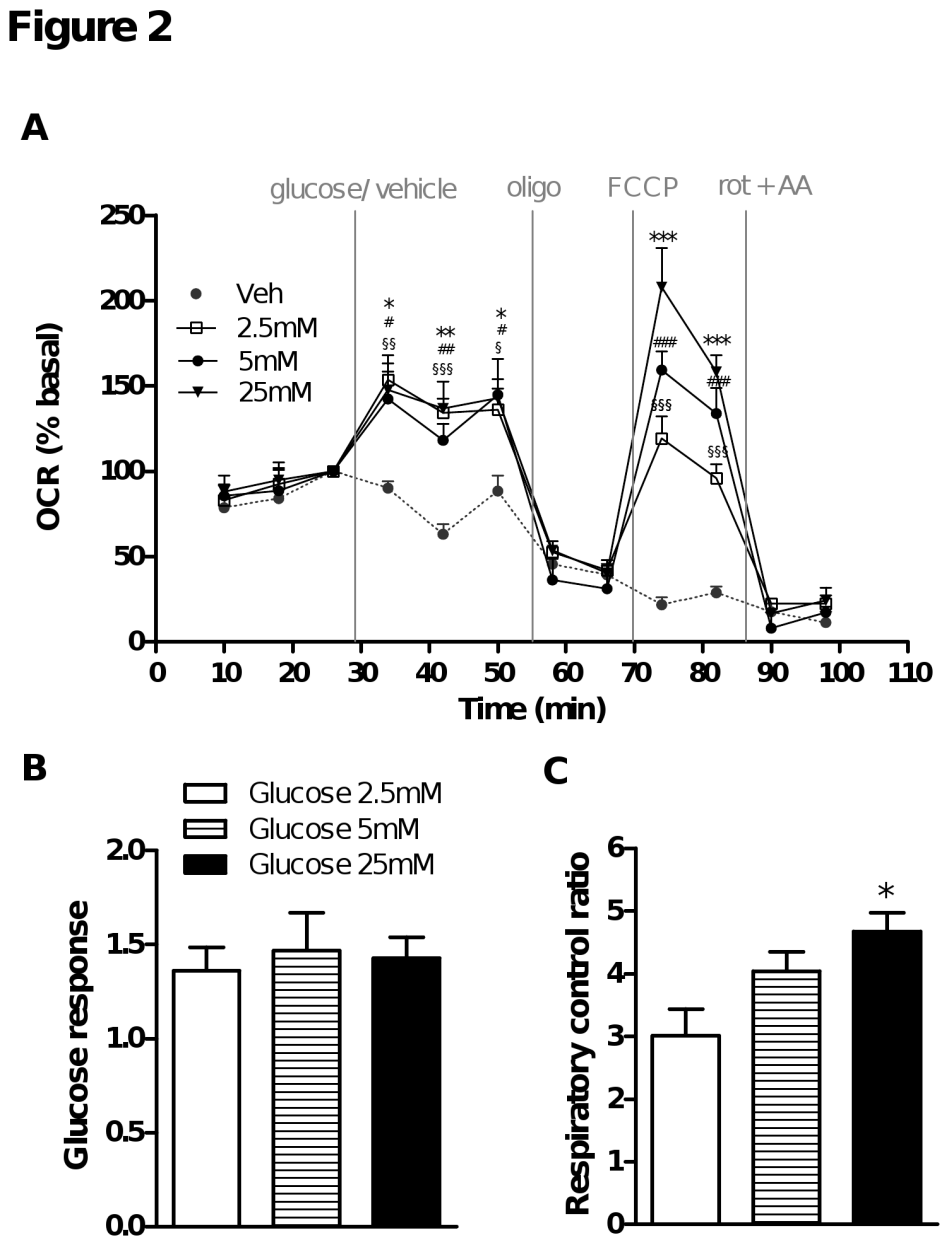
Respiration parameters measured in WT striatal neurons as a function of glucose concentration. On the day of the respiration experiments, medium was replaced with substrate-free KHB and cells were incubated for 1 h at 37°C. OCR measurements began 10 min after cell plate installation in the Seahorse analyser. A) After measuring the initial OCR in the absence of substrates, various concentrations of glucose in KHB (vehicle; Veh) were injected (final concentrations: 0, 2.5, 5 or 25 mM). OCR was then measured in the presence of substrate and following successive injections of oligomycin (oligo), FCCP, then rotenone and antimycin A (rot + AA) at the indicated times. Data are expressed as the percentage of the basal value measured at the time point 3 (four replicates). § p < 0.05, §§ p < 0.01, §§§ p < 0.001 (0 mM vs. 2.5 mM glucose); # p < 0.05, ## p < 0.01, ### p < 0.001 (0 mM vs. 5 mM glucose); * p < 0.05, ** p < 0.01, ***p < 0.001 (0 mM vs. 25 mM glucose). Respiratory parameters were derived from OCR measured during each of these phases; B) glucose response = [basal glucose rate (time point 6) / basal free substrate rate (time point 3)]; C) respiratory control ratio = [FCCP rate (time point 9) / oligomycin rate (time point 8)]. * p < 0.05 compared to 2.5 mM glucose.

In first experiments, the medium used to measure respiration contained 25 mM glucose to mimic their original culture conditions. This is a high, supra-physiological concentration compared to the glucose concentrations found in brain that are estimated to be around 2.5 mM in rats [31]. To check whether there was any difference in the ability of HD striatal neurons to use more physiological levels of glucose as well as various other substrates to support oxidative phosphorylation, we performed a series of experiments adding specific substrates to cells incubated in KHB. Different concentrations of glucose were injected (final concentrations of 2.5 - 25 mM) followed by oligomycin, FCCP, and rotenone + antimycin A injections (Figure 2A). In the absence of glucose, neurons were able to maintain basal respiration but they were not able to respond to FCCP stimulation (Figure 2A). All concentrations of glucose between 2.5 and 25 mM induced a similar increase in basal OCR (Figure 2B) but even though basal respiration was similar in the presence of physiological and supra-physiological glucose concentrations (Figure 2B), FCCP-stimulated respiration and RCR were glucose-concentration dependent (Figure 2C). These results suggest that the physiological glucose concentration found in brain, although sufficient to support respiration under basal conditions (low energy demand) may be limiting under conditions of high respiratory demand. 

### Neurons can use lactate and pyruvate to support respiration

**Figure 3:**
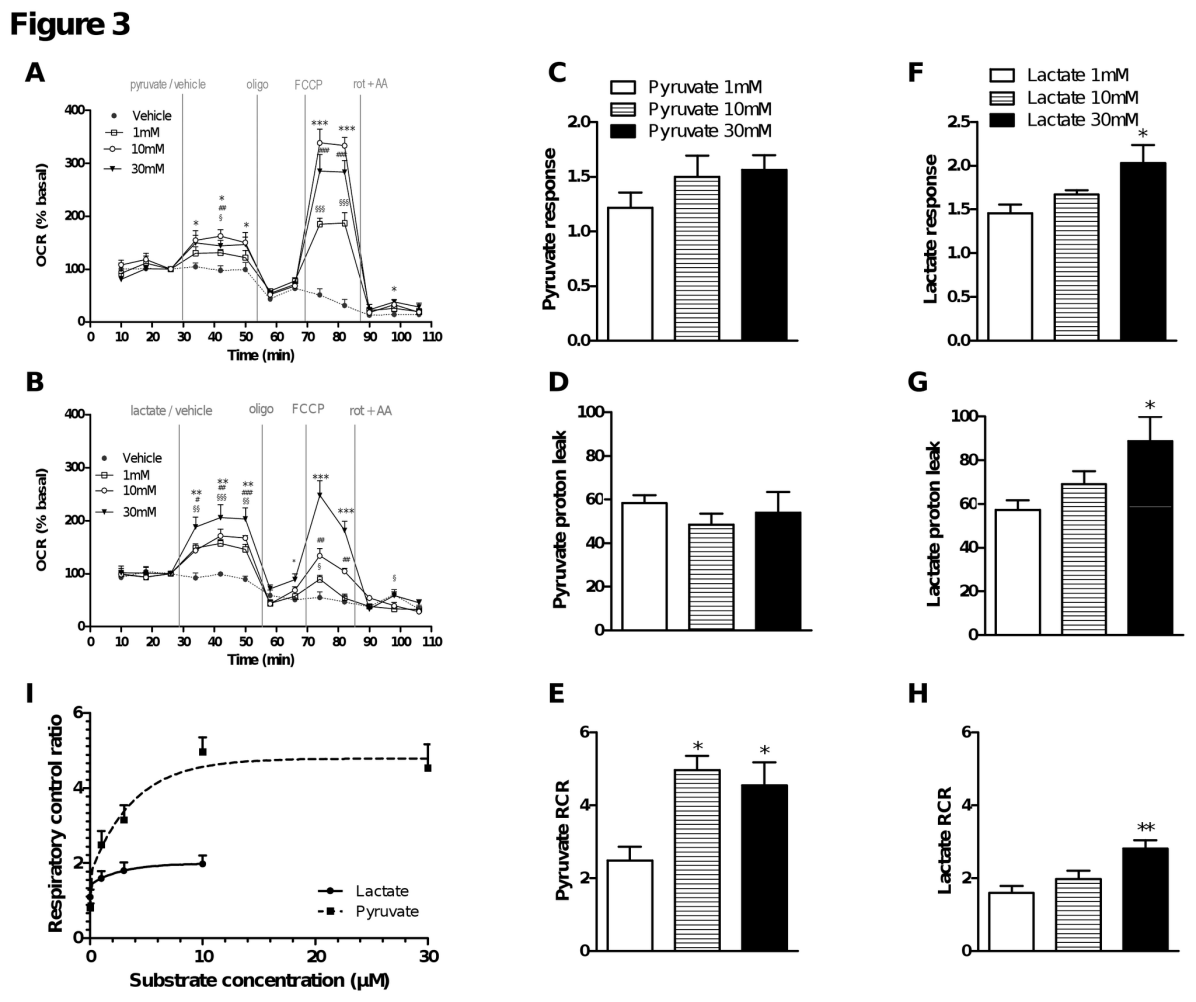
Striatal neurons can use alternative substrates to glucose to support respiration. On the day of the respiration experiments, medium was replaced with substrate-free KHB and cells were incubated as described in Figure 2. After measuring the initial OCR in the absence of substrates, various concentrations of pyruvate (A) or lactate (B) in KHB (vehicle; Veh) were injected (final concentrations: 0, 1, 10 or 30 mM). OCR was then measured in the presence of substrate and following successive injections of oligomycin (oligo), FCCP, then rotenone and antimycin A (rot + AA) at the indicated times. Data are expressed as the percentage of the basal value measured at time point 3 (four replicates). § p < 0.05, §§ p < 0.01, §§§ p < 0.001 (0 mM vs. 1 mM pyruvate or lactate); # p < 0.05, ## p < 0.01, ### p < 0.001 (0 mM vs. 10 mM pyruvate or lactate); * p < 0.05, ** p < 0.01, ***p < 0.001 (0 mM vs. 30 mM pyruvate or lactate). Respiratory parameters were derived from the various phases following pyruvate (C-E) or lactate (F-H) injection: substrate response = [basal pyruvate or lactate rate (time point 6) / basal substrate free rate (time point 3)]; proton leak rate (time point 7); respiratory control ratio = [FCCP rate (time point 9) / oligomycin rate (time point 8)]. * p < 0.05, ** p < 0.01 compared to 1 mM pyruvate (C-E) or lactate (F-H). I). Respiratory control ratio expressed as a function of pyruvate (1, 3, 10 or 30 mM) or lactate (1, 3 or 10 mM) concentration.

Increasing evidence supports the idea that glucose is not the sole energy substrate for neurons in the brain. Oxidative substrates such as pyruvate or even lactate provided by glial cells could be supplementary fuel for neurons [32]. Results presented in Figure 3 reinforced this hypothesis. WT neurons were incubated for 1 hour in KHB medium lacking metabolic substrates then provided with various concentrations of pyruvate (Figure 3A) or lactate (Figure 3B). Then respiratory parameters were derived following injection of oligomycin, FCCP, and rotenone + antimycin A. As observed in the previous study assaying the response to glucose, in the absence of substrates, neurons were able to maintain basal respiration but were not able to respond to FCCP stimulation (Figure 3A; 3B). Addition of either pyruvate or lactate increased basal respiration as previously seen with glucose and restored the neurons’ capacity to respond to FCCP stimulation (Figure 3A, 3B). In the case of pyruvate, a similar basal respiration rate was detected over the concentration range tested (1-30 mM) but maximal respiration and RCR reached a plateau between 1 and 10 mM (Figure 3A, 3C, 3E). Addition of 1 or 10 mM lactate resulted in a similar basal respiration rate, which was significantly increased in the presence of 30 mM lactate (Figure 3B-F). FCCP stimulated respiration was also supported by lactate but to a lesser extent than pyruvate. This can be explained by the observation that 30 mM lactate induced a significant increase in proton leak respiration in addition to ATP synthesis-driven respiration (Figure 3G). This was not the case for 30 mM pyruvate (Figure 3D). Because 30 mM lactate increased mitochondrial proton leak, potentially disturbing mitochondrial function, this concentration was not considered in further analysis of RCR as a function of pyruvate and lactate concentrations (Figure 3I). We observed a concentration-dependent increase in RCR for both lactate and pyruvate, plateauing at 10 mM for pyruvate. Comparing the RCR between 10 mM pyruvate and lactate shows that pyruvate is clearly a better substrate, most likely due to the fact that lactate must first be converted to pyruvate.

### Comparison of respiratory parameters in WT and HD striatal neurons in the presence of a physiological glucose concentration

**Figure 4:**
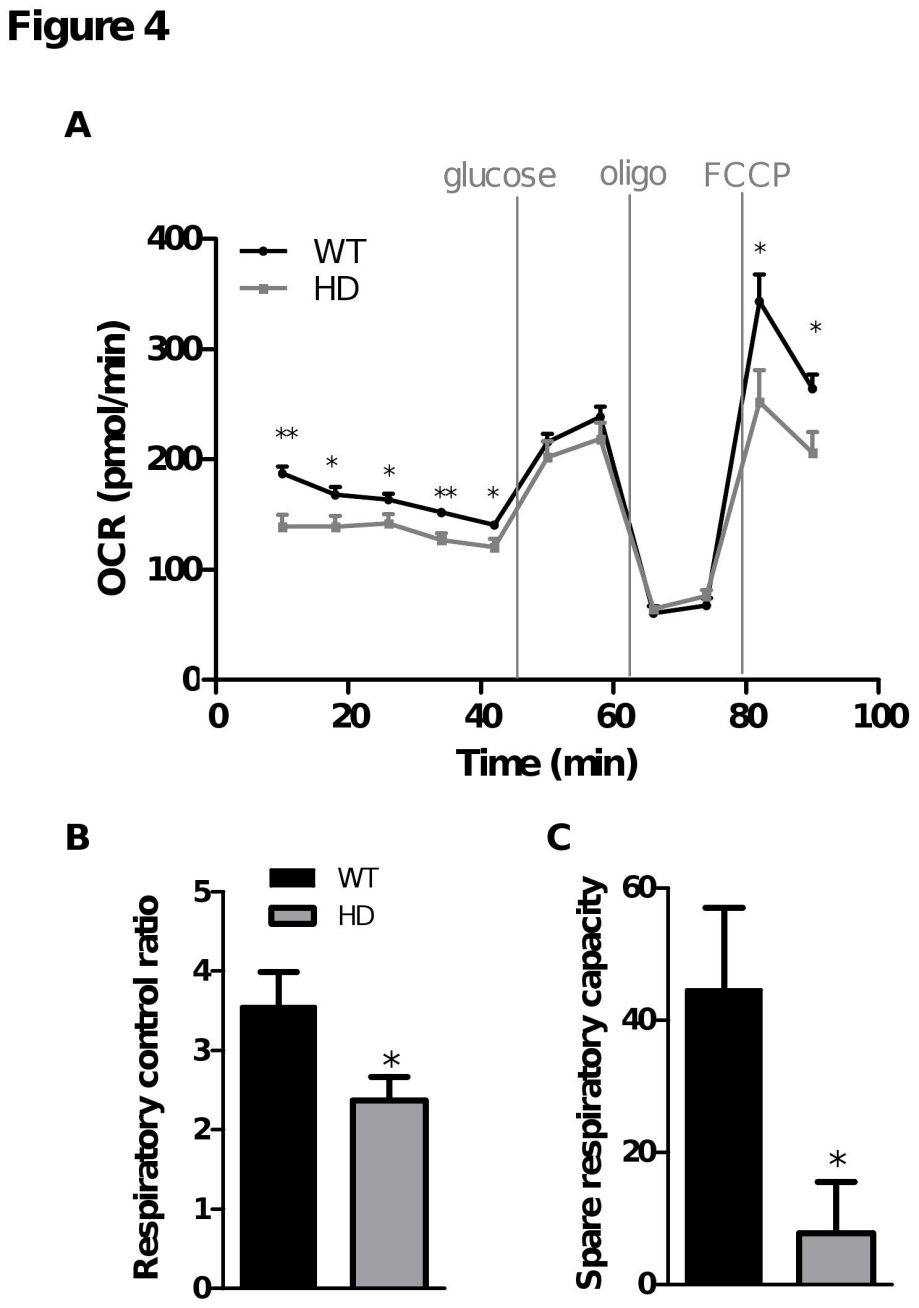
OCR measurements performed using a physiological glucose concentration revealed respiratory deficits in HD striatal neurons. OCR measurements were performed as described in Figures 2. A) OCR measurements were taken following injection of glucose (final concentration, 2.5 mM), oligomycin (oligo) then FCCP at the indicated times. Data shown are mean ± SEM from a representative experiment. Respiratory parameters were derived from the various phases following glucose injection (B-C): respiratory control ratio = [FCCP rate (time point 10) / oligo rate (time point 9)]; glucose spare respiratory capacity = [FCCP rate (time point 10) – glucose rate (time point 7)]. Data shown are mean ± SEM from all replicates (six replicates in each of three independent experiments). * p < 0.05, ** p < 0.01 comparing HD vs. WT.

To see whether there is any difference in the capacity of WT and HD striatal neurons to use physiological glucose concentrations, OCR was measured in KHB as shown in Figure 2. First, OCR was measured after one hour of substrate deprivation. Although there were some differences noted during the equilibration period seen in some experiments, overall, there were little or no significant differences detected between the two genotypes following more than one hour in this challenging condition, suggesting that intracellular substrates and neurons’ capacity to mobilize them were similar between WT and HD striatal neurons (Figure 4A). Furthermore, as observed in Figure 2, addition of 2.5 mM glucose increased OCR to a similar basal level in both WT and HD striatal neurons (Figure 4A) and oligomycin injection induced a similar reduction in OCR and in proton leak respiration in the two genotypes (Figure 4A). These results show that there is no underlying mitochondrial dysfunction in HD striatal neurons or their ability to use glucose to support basal respiration and ATP synthesis. By contrast, when respiration was stimulated with FCCP, OCR deficits associated with lower RCR and spare respiratory capacity were detected in HD striatal neurons compared to WT (Figure 4B, 4C), suggesting mitochondrial respiratory chain dysfunction and/or a defect in glucose metabolism that generates pyruvate (glycolysis) under stimulated conditions. 

### Respiration in WT and HD striatal neurons supported by pyruvate or lactate

**Figure 5:**
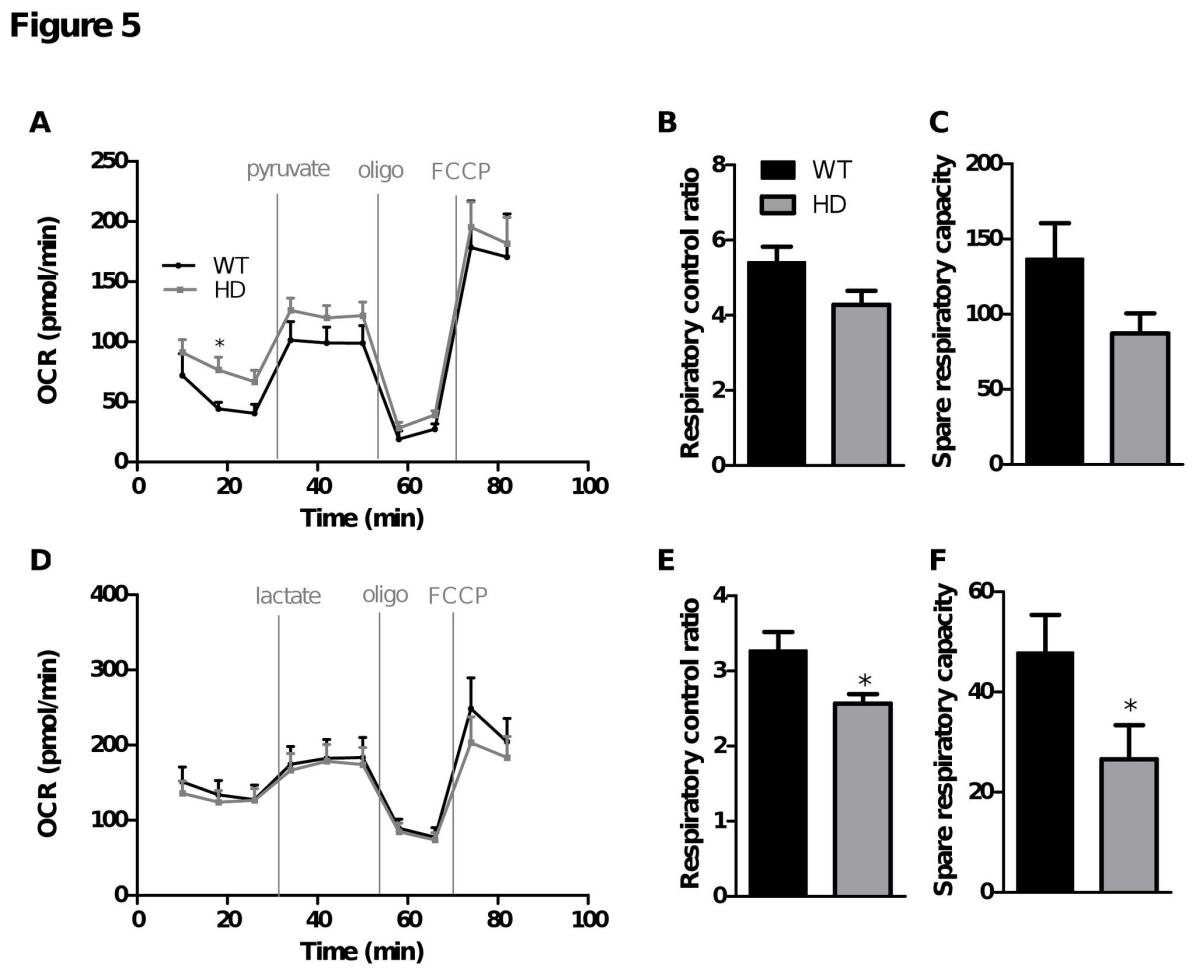
Comparison of HD and WT striatal neurons’ ability to use pyruvate and lactate in the absence of glucose. OCR measurements were performed as described in Figure 3. OCR measurements were taken following injection of 10 mM pyruvate (A) or lactate (D), oligomycin (oligo) and FCCP (2 µM) at the indicated times. Data shown are mean ± SEM from a representative experiment (six or ten replicates for pyruvate or lactate, respectively). Respiratory parameters were derived from the various phases following pyruvate (B-C) or lactate (E-F) injection: respiratory control ratio = [FCCP rate (time point 9) / oligo rate (time point 8)]; spare respiratory capacity = [FCCP rate (time point 9) – pyruvate or lactate rate (time point 6)]. Data for respiratory parameters are mean ± SEM calculated from all replicates (three independent experiments). * p < 0.05 comparing HD vs. WT.

We also evaluated HD and WT striatal neurons’ capacity to use alternative substrates, pyruvate and lactate according the protocol set up in Figure 3. The concentration chosen for this exploration was 10 mM for each substrate, which was the concentration inducing the maximal OCR response under stimulated conditions without causing proton leak. As previously shown in Figure 4, after more than one hour of substrate deprivation, OCR was similar between WT and HD neurons (Figure 5A, 5D). Pyruvate addition induced a significant increase in basal OCR and restored neurons’ capacity to respond to FCCP (Figure 5A). No significant differences were detected in HD striatal neurons compared to WT (Figure 5A-C). Lactate addition induced a similar increase in basal OCR as pyruvate, which was again not different between WT and HD neurons (Figure 5D). Even though respiration in the presence of 10 mM lactate was similar or only slightly lower in HD neurons, the resulting RCR and spare respiratory capacity appeared to be significantly lower in FCCP-stimulated HD neurons compare to WT (Figure 5E-F).

**Figure 6:**
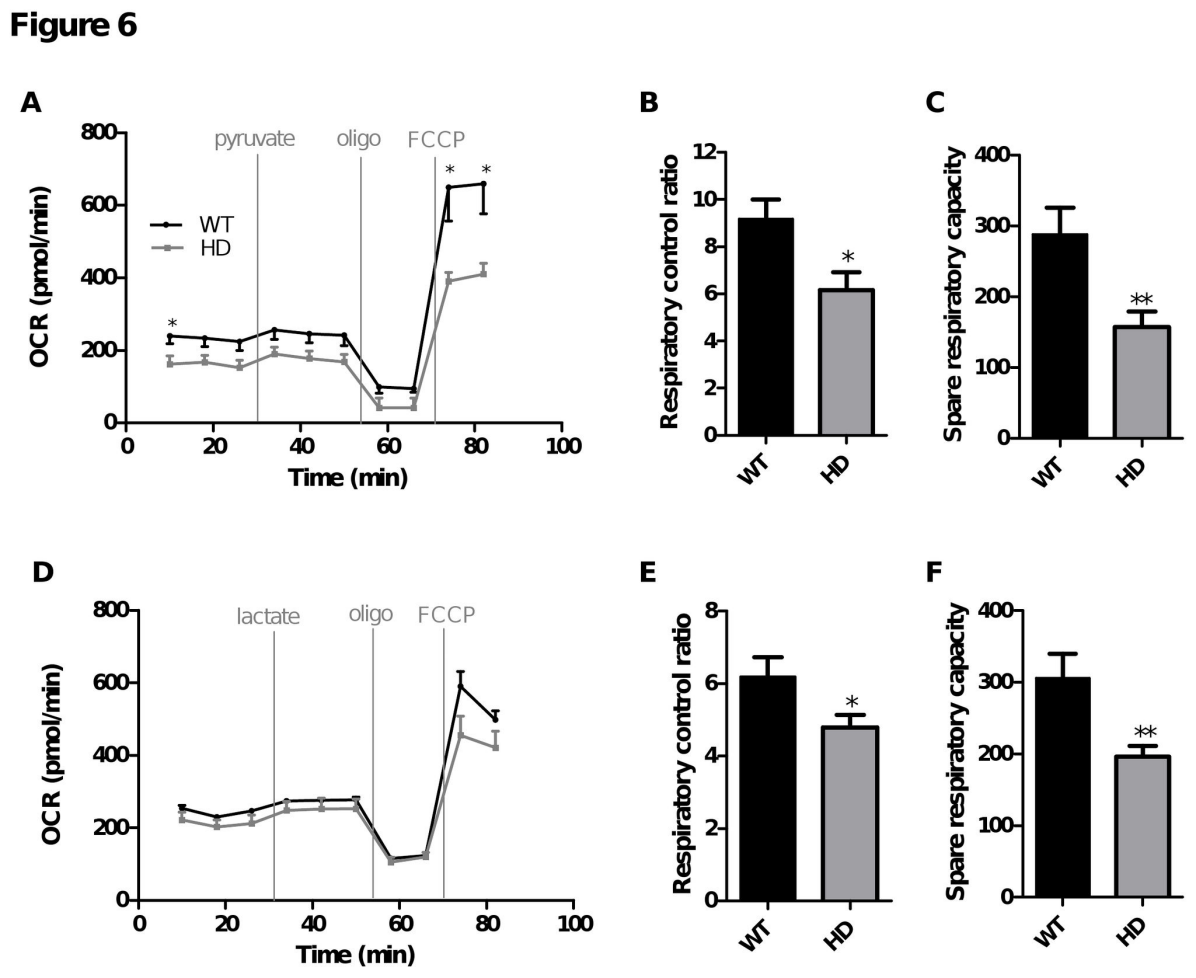
HD striatal neurons have a lower capacity to use glucose under high respiratory demands, even when supplemented with pyruvate or lactate. Striatal neurons were cultivated for 7 days as described in Figure 1. One hour before the start of the respiration experiments, medium was replaced with substrate-free KHB complemented with 2.5 mM glucose. OCR measurements were performed as described in Figure 3. OCR measurements following injection of 10 mM pyruvate (A) or lactate (D), oligomycin (oligo) and FCCP (2 µM) at the indicated times. Data shown are mean ± SEM from a representative experiment (five or four replicates for pyruvate or lactate, respectively). Respiratory parameters were derived from the various phases following pyruvate (B-C) or lactate (E-F) injection: respiratory control ratio = [FCCP rate (time point 9) / oligo rate (time point 8)]; spare respiratory capacity = [FCCP rate (time point 9) - pyruvate or lactate rate (time point 6)]. Data for respiratory parameters are mean ± SEM calculated from all replicates (three independent experiments). * p < 0.05, ** p < 0.01 comparing HD vs. WT.

To see how neurons respond to a mixture of metabolic substrates, OCR was measured in WT and HD striatal neurons in the presence of 2.5 mM glucose followed by addition of 10 mM lactate or pyruvate (Figure 6). Neither pyruvate nor lactate increased basal OCR further when neurons were already supplied with a physiological glucose concentration (Figure 6A-D), suggesting that glucose alone was sufficient to support basic neuron energy requirements. Moreover, this result confirmed that under basal conditions, respiration is probably strongly controlled by the rate of mitochondrial ATP turnover or proton leak and is not limited by the rate of substrate oxidation. FCCP injection induced a large increase in OCR in the two conditions tested. In the presence of glucose and pyruvate, RCR was higher compared to glucose or pyruvate alone (9.2 ± 0.8, 5.4 ± 0.4, 3.5 ± 0.4; glucose + pyruvate RCR, pyruvate RCR, glucose RCR, respectively; mean ± SEM). Similar results were obtained with lactate, with higher RCR in the presence of glucose and lactate compared to glucose or lactate alone (6.2 ± 0.5, 3.3 ± 0.2, 3.5 ± 0.4; glucose + lactate RCR, lactate RCR, glucose RCR, respectively; mean ± SEM). These results suggest that in the presence of a physiological glucose concentration, maximal respiration can be improved when alternative substrates such as pyruvate or lactate are available. However, it can be seen that FCCP-stimulated OCR was significantly lower in HD compared to WT striatal neurons when supplied with either pyruvate or lactate in addition to a physiological glucose concentration (Figure 6A-D). This shows that the respiratory defects observed under stress conditions in the presence of physiological concentration of glucose (Figure 4) were not completely compensated by the presence of alternative substrates, such as pyruvate or lactate. 

### Comparison of ECAR in WT and HD primary striatal neurons

**Figure 7:**
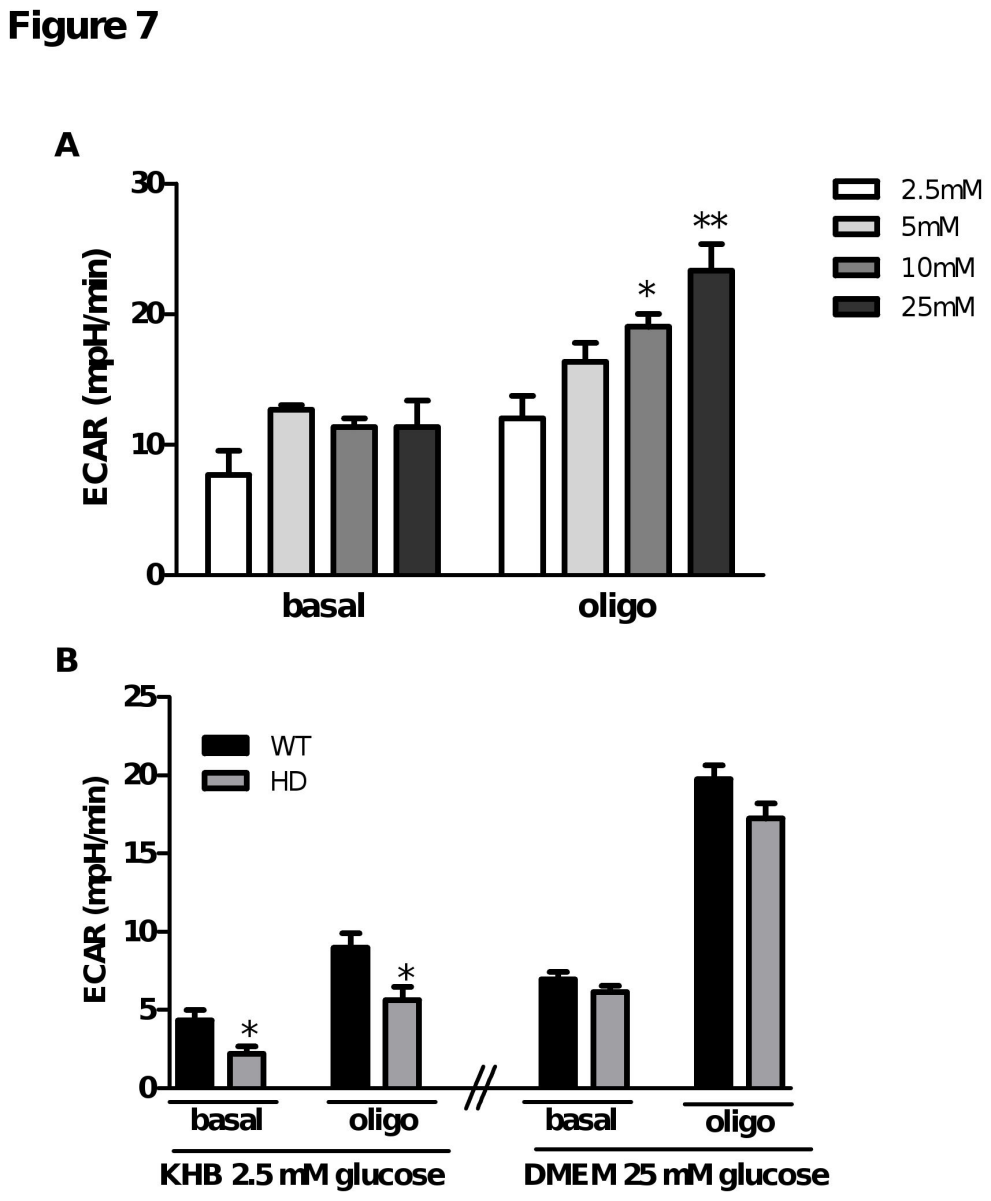
HD striatal neurons have a lower capacity to use anaerobic glycolysis in the presence of high and low glucose concentrations. Striatal neurons were cultivated for 7 days in Neurobasal™ medium containing 25 mM glucose and complemented with B27 and pyruvate (1 mM). A) On the day of the respiration experiments, medium was replaced with substrate-free KHB and WT cells were incubated for 1 h at 37°C. ECAR was measured after injection of different concentrations of glucose then oligomycin. Data are mean ± SEM and derived from the same experiment presented in Figure 2. * p < 0.05, ** p < 0.01 vs. 2.5 mM glucose. B) On the day of the respiration experiments, cells were incubated in 2.5 mM glucose in KHB medium or bicarbonate- and HEPES-free DMEM containing 25 mM glucose complemented with pyruvate (1 mM) for 1 h at 37°C. ECAR was measured under basal conditions and after oligomycin stimulation. Data are mean ± SEM from all independent experiments (2.5 mM glucose: five replicates in each of five experiments, n=25; 25 mM glucose: eleven replicates in each of seven experiments, n=77). . * p<0.05, ** p < 0.01 comparing WT vs. HD.

To investigate if OCR deficits observed in the presence of 2.5 mM glucose in HD primary striatal neurons were associated with a defect in anaerobic glycolysis, we measured ECAR in HD and WT striatal neurons. First, we observed under basal conditions that ECAR in WT striatal neurons remained stable regardless of the glucose concentration used (Figure 7A). After oligomycin injection, the inhibition of ATP synthesis by the respiratory chain resulted in ECAR activation as previously described [29]. Interestingly, this activation was glucose concentration dependent (Figure 7A). Because FCCP increases H^+^ extrusion, ECAR could not be used as a measure of glycolysis under uncoupling conditions; therefore, data obtained after FCCP injection was not analyzed. Then, ECAR was measured in WT and HD primary striatal neurons incubated either in rich substrate DMEM or in KHB containing 2.5 mM glucose (Figure 7B). Under basal conditions, no significant difference was observed between WT and HD neurons. In contrast, oligomycin-stimulated ECAR as an indicator of glycolysis was significantly lower in HD neurons both in rich and low glucose medium, suggesting a specific defect in their maximal anaerobic glycolysis capacity. At the same time, intracellular pH measured using 2',7'-Bis-(2-Carboxyethyl)-5-(and-6)-Carboxyfluorescein (BCECF Invitrogen, 0.3µM) was similar in WT and HD neurons suggesting no difference in lactate release between the two genotypes (data not shown).

### Comparison of respiration parameters in WT and HD cortical neurons

**Figure 8:**
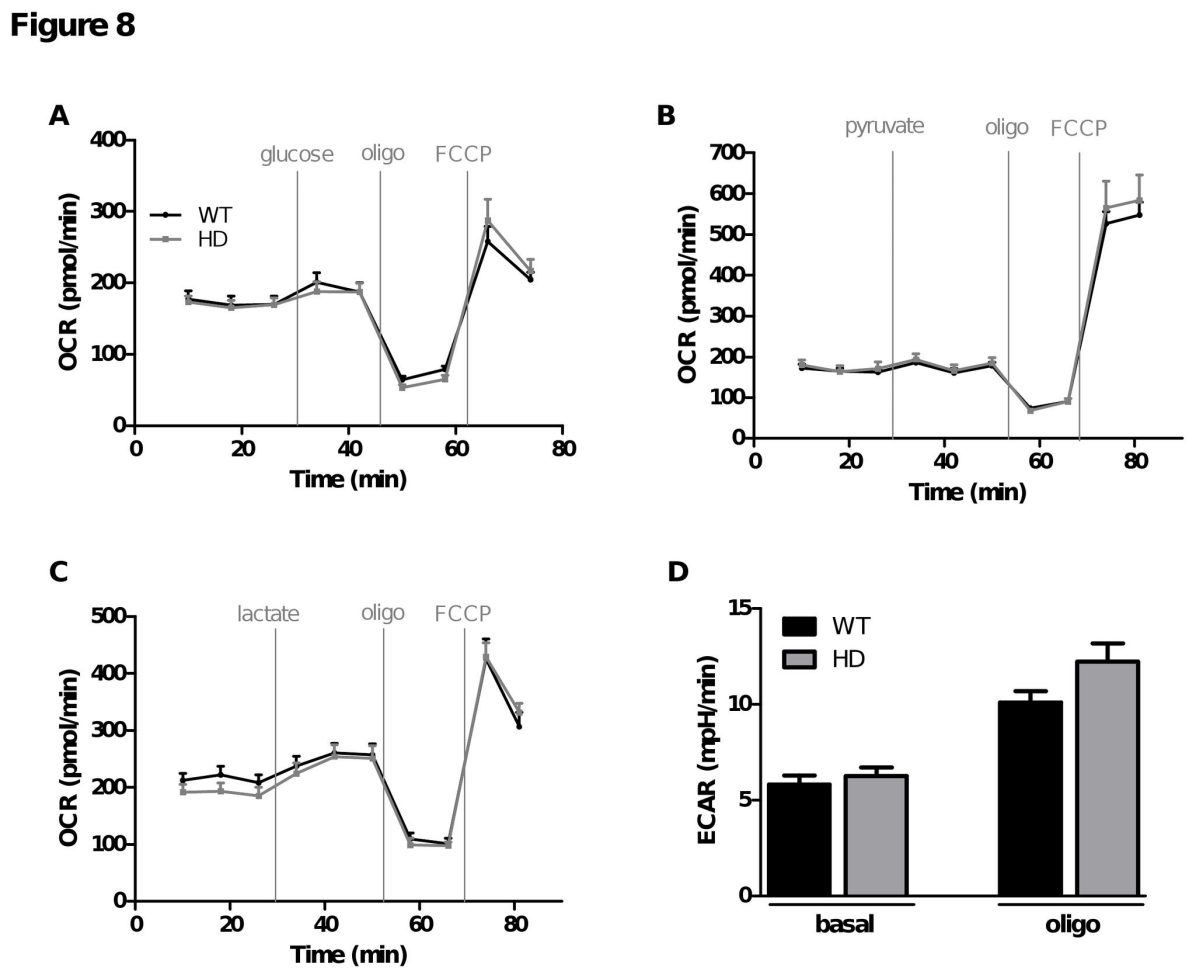
HD cortical neurons had no metabolic deficits regardless of the substrate supplied. HD and WT cortical neurons were cultivated for 7 days in Neurobasal™ medium complemented with B27 and pyruvate (1 mM). One hour before the start of respiration experiments, medium was replaced with substrate-free KHB and incubated at 37°C before being placed in the Seahorse analyser. OCR measurements were performed as described in Figures 2 and 3. OCR measurements following injection of (A) 2.5 mM glucose, (B) 10 mM pyruvate or (C) 10 mM lactate followed by oligomycin (oligo) and FCCP (2 µM) at the indicated times. D) ECAR was measured in the presence of 2.5 mM glucose under basal conditions and after oligomycin stimulation. Data shown in panels A-C are from representative experiments while data in panel D is the mean ± SEM calculated from all replicates (eight replicates for each substrate in each of two independent experiments)..

To investigate if the deficits seen in HD striatal neurons were common to all types of neurons, we performed similar experiments using primary cortical neurons, a brain region affected later in Huntington disease [33]. In contrast to striatal neurons, no respiratory deficits were detected in the presence of 2.5 mM glucose alone under basal or uncoupling conditions (Figure 8A) and RCR was similar between HD and WT cortical neurons. Additionally, in the presence of glucose (2.5 mM) and pyruvate (10 mM) or lactate (10 mM), no defect was detected in HD cortical neurons in any of the respiratory parameters (Figure 8B-C). ECAR measurements were also performed in presence of 2.5 mM glucose in primary cortical neurons and results did not show any defects in HD compared to WT (Figure 8D). 

## Discussion

In this study, we measured the ability of various substrates to support mitochondrial respiration in intact primary striatal and cortical neurons obtained from WT and HD rat embryos. These studies were designed to detect potential metabolic differences in HD neurons that could contribute to the pattern of neurodegeneration found in HD. We detected no specific mitochondrial respiratory chain defects in either cortical or striatal neurons from HD rats. By contrast, we found defects in glycolysis that are present even in cells prepared from embryos, but only in primary striatal and not cortical neurons derived from this rat HD model even though mHtt aggregates are present in both types of neurons (see Figure S1). 

As previously mentioned, most of the studies exploring mitochondrial defects in HD models were done using brain extracts containing heterogeneous mixtures of neuronal and glial mitochondria, which may have precluded clear conclusions. In this study, we monitored metabolic rates in specific primary neuronal subpopulations (cortical or striatal) attached to the culture plates after 7 days in culture using extracellular flux measurement technology [29]. This allowed us to measure OCR in intact cells including all mitochondria populations located in neuronal cell bodies as well as neurites. Indeed, mitochondria localized in dendrites, axon shafts and presynaptic terminals play a key role in neuronal metabolism and in neuroplasticity [34] and these structures are largely left behind when neurons are detached from their culture surface for respiration measurements; this mitochondrial fraction is important especially when considering that axonal transport is disturbed in HD [35]. In addition, these studies highlighted the importance of alternate substrates to support neuronal respiration along with physiological glucose concentrations found in brain, particularly when respiration demand is high. The normal plasma glucose concentration is around 4.5 mM; however, the glucose concentration in the CNS is only about 2.5 mM [31,36]. Neurobasal™ medium, commonly used to culture primary neurons contains 25 mM glucose, a supra-physiological concentration. We found that glucose dose-dependently increased neuronal spare respiratory capacity and RCR and that 2.5 mM glucose was clearly suboptimal to support neurons under high respiratory demand. We then found that both lactate and pyruvate were individually able to support mitochondrial respiration in intact neurons and that these alternative substrates could provide additional respiratory capacity beyond that provided by CNS levels of glucose when neurons were challenged with an uncoupling agent. These results highlight the importance of neuronal glial coupling whereby glucose utilization by astrocytes results in the release of lactate that can then be taken up and used by neurons to support respiration [37-39]. Lactate conversion to pyruvate may take place preferentially in nerve terminals depending on differential distribution of LDH isoforms found in neuron cell bodies and terminals [40]. Furthermore, MCT1 transporters have recently been implicated in lactate exchange between oligodendrocytes and neuronal axons [41]. Interestingly, we observed an additive effect of glucose and pyruvate on maximal, FCCP-stimulated OCR (refer to maximal OCR in Figures 4, 5, 6). These results support the existence of a glucose dependent but pyruvate independent system transferring electrons to the respiratory chain in neurons. Indeed, the glycerol 3-phosphate shuttle directly transfers electrons to coenzyme Q in the mitochondrial inner membrane. This shuttle, active in neurons, is regulated by NADH/NAD+ and calcium and depends on the first step of glycolysis [42-44]. 

When HD striatal neurons were incubated in substrate-rich media containing a supra-physiological glucose concentration, no respiratory deficits were detected. These results are consistent with previous results obtained in HD knock-in mouse primary striatal neurons [25] where mitochondrial respiration was monitored in medium containing 15 mM glucose. In addition to these results, we now show that when the respiration demand is high, HD striatal neurons exhibit respiratory deficits when supplied with 2.5 mM glucose either alone or when supplemented with lactate or pyruvate. When pyruvate was the sole substrate present in the assay media, no significant respiratory deficits were observed in HD striatal neurons. These results argue for an alteration in glucose uptake and/or in glycolysis in HD rather than any defects in the respiratory chain itself. This hypothesis is further supported by the fact that deficits were only detected in the presence of physiological glucose concentrations but not when glucose was abundant. Moreover, because glucose defects in OCR were not compensated by pyruvate addition, it appears that a glucose-dependent but pyruvate independent electron transfer pathway is down regulated in HD primary striatal neurons. Extracellular acidification is a valid indicator of the anaerobic glycolysis rate (metabolic pathway converting glucose into lactate) and a valuable tool for analysing cellular bioenergetics [29,45]. Here, we observed that ECAR deficits were detected in HD striatal neurons both when incubated in culture medium containing a supra-physiological glucose concentration but also in KHB containing 2.5 mM glucose. Taken together, the OCR and ECAR data strongly support the hypothesis that striatal glycolysis deficits occur early in HD pathogenesis. Dysregulation in glycolysis has been reported in several studies in animal and cellular HD models or in patients [20,46-48]. Additionally, Powers and colleagues reported a preserved mitochondrial oxidative metabolism in early HD patients with striatal atrophy, indicating that defects in respiratory chain enzymes observed in post mortem brain are either not sufficient to explain oxidative phosphorylation impairments or are not present early in the time course of the disease [20]. They also reported in the same early HD patients a decrease in cerebral glucose metabolism indicating a selective impairment of striatal glycolytic metabolism [20]. Very recently, Zala et al. showed that the glycolytic enzyme, GAPDH, is located on neuron vesicles and that local glycolysis powers vesicular fast axonal transport. Additionally, the authors demonstrated that huntingtin is a scaffold that joins GAPDH to these vesicles, suggesting that mutations in huntingtin could perturb glycolysis-generated ATP necessary for vesicle motility [49]. 

Interestingly, in our study, even though mHtt aggregates are present in both cell types, metabolic defects were only detected in embryonic striatal neurons and not in cortical neurons, a brain region affected later in Huntington disease progression. Indeed, cortical neurons showed neither OCR deficits nor ECAR alterations in the presence of a physiological concentration of glucose. These results provide additional insight into the mechanisms of selective striatal neurodegeneration and into the relative metabolic vulnerability of different cellular populations to mHtt toxicity. In the light of the present study, it might be argued that striatal neurons seem to have different metabolic requirements compared to cortex and may have a reduced capacity to manage substrate deprivation. Indeed, we can observe in Figures 4A and 8A that glucose injection did not have the same impact on basal OCR in striatal and cortical neurons. In striatal neurons a significant increase of 31.5 ± 5.8% was observed in response to glucose injection; for cortical neurons, which had a higher basal OCR in the absence of glucose, the response was only 9 ± 2.9%. 

In summary, early glycolysis defects are found specifically in HD striatal neurons. These subtle defects, observed only with levels of glucose found in brain, may only have adverse consequences after prolonged stress or in combination with other age-related declines in metabolism, explaining why neurodegeneration only becomes evident in HD gene carriers in middle age. These results can be related to new insights in pre-symptomatic carriers of apolipoprotein E4, who show reduced cerebral glucose metabolism even before Aβ aggregation and decades before the onset of AD pathology [50,51]. These brain functional abnormalities in neurodegenerative disease gene carriers argue for early prevention therapies, decades before the onset of cognitive or motor symptoms. 

## Supporting Information

Protocol S1 **Methods for assessing mhtt expression in primary neuronal cultures.**
(DOC)

Figure S1 **Confirmation of mhtt expression in primary neuronal cultures.** Primary striatal and cortical neurons from HD rats express mhtt. SDS-insoluble and thus aggregated proteins were trapped on a nitrocellulose membrane and probed with a polyQ-specific antibody. The presence of aggregated polyQ-containing protein in primary striatal and cortical cultures from HD rat embryos but not their WT littermates indicates the expression of aggregated forms of mhtt in these neurons. Each dot is the extract from a separate neuronal culture, prepared from an individual WT or HD embryo.
(TIF)
