# Supplementary material for: Early Deficits in Glycolysis Are Specific to Striatal Neurons from a Rat Model of Huntington Disease
Source: PLoS One. 2013 Nov 26;8(11):e81528. doi: 10.1371/journal.pone.0081528 (PMC3841140; doi:10.1371/journal.pone.0081528)
Supplement: Protocol S1 — Methods for assessing mhtt expression in primary neuronal cultures. (DOC) [file pone.0081528.s001.doc]

Striatal and cortical neurons prepared from individual HD and WT embryos were cultured in 6-well plates at a density of 1,000,000 cells/well for cortex and 2,000,000 cells/well for striatum. After 7 days in vitro, cells were harvested by trypsinization (1 ml of 0.25 % trypsin with EDTA (Gibco) per well) and centrifugation (10 min at 400xg). Proteins were extracted from cell pellets by incubation in 100 µl RIPA buffer (50 mM Tris-HCl, pH 8.0, 150 mM NaCl, 1 % IGPAL (NP-40 substitute), 0.5 % sodiumdesoxychelate, 0.1 % SDS) containing 4 % cOmplete ULTRA Protease Inhibitor-Cocktail with EDTA (Roche) for 30 min at 4 °C and subsequent centrifugation for 15 min at 4 °C at 16.400xg. Supernatant was collected and protein content for each sample was measured using a Bradford assay. The samples were stored at -80 °C until further use. For the analysis of mhtt expression in these samples, we performed a filter trap assay [1] optimized for the detection of mhtt. The method is based on the SDS-insolubility of mhtt aggregates, which are retained when filtered through a nitrocellulose membrane. For this purpose, 20 µg of lysate protein was mixed with SDS (final concentration 2 %) and filtered using a dot blotter (Dot blotter SRC 96 D, S&S Minifold I, Schleicher & Schuell, Germany). The membrane (Protran Nitrocellulose Membrane, Whatman) was afterwards probed with 1C2 anti-polyQ-antibody (MAB1574, Millipore, 1:2000) overnight at 4 °C and on the next day incubated for 1 hour at room temperature with the secondary, horse-radish-peroxidase-linked anti-mouse IgG antibody (NA931, Amersham Biosiences, 1:2500). Chemiluminescence was created using ECL Western Blotting Detection Reagent (Amersham Biosciences) and detected with the Odyssey FC (LI-COR Biosciences), showing aggregated forms of polyQ-containing protein (mhtt) (Fig S1).

1. Wanker EE, Scherzinger E, Heiser V, Sittler A, Eickhoff H, et al. (1999) Membrane filter assay for detection of amyloid-like polyglutamine-containing protein aggregates. Methods Enzymol 309: 375-386.
