# Supplementary figures and images for: Early Deficits in Glycolysis Are Specific to Striatal Neurons from a Rat Model of Huntington Disease
Source: PLoS One. 2013 Nov 26;8(11):e81528. doi: 10.1371/journal.pone.0081528 (PMC3841140; doi:10.1371/journal.pone.0081528)

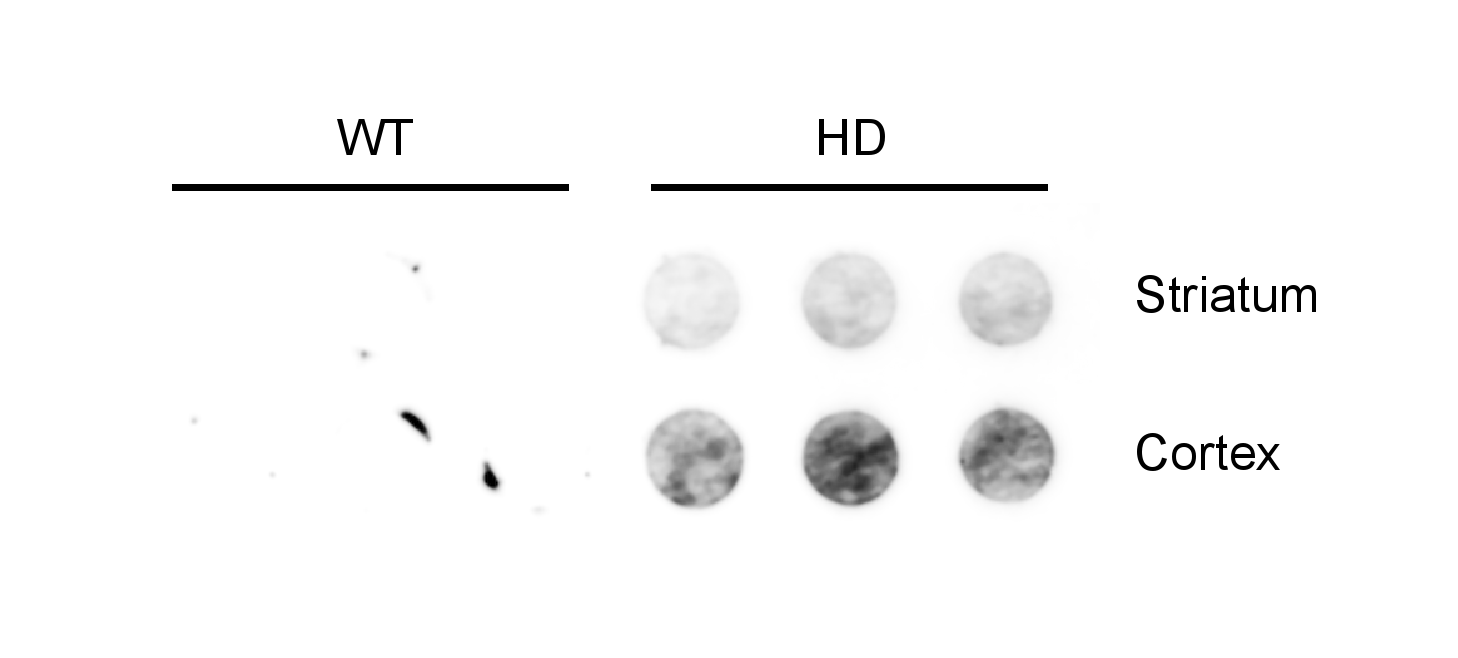

Supplement: Figure S1 — Confirmation of mhtt expression in primary neuronal cultures. Primary striatal and cortical neurons from HD rats express mhtt. SDS-insoluble and thus aggregated proteins were trapped on a nitrocellulose membrane and probed with a polyQ-specific antibody. The presence of aggregated polyQ-containing protein in primary striatal and cortical cultures from HD rat embryos but not their WT littermates indicates the expression of aggregated forms of mhtt in these neurons. Each dot is the extract from a separate neuronal culture, prepared from an individual WT or HD embryo. (TIF) [file pone.0081528.s002.tif]
